# Benign Effect of Extremely Low-Frequency Electromagnetic Field on Brain Plasticity Assessed by Nitric Oxide Metabolism during Poststroke Rehabilitation

**DOI:** 10.1155/2017/2181942

**Published:** 2017-09-12

**Authors:** Natalia Cichoń, Piotr Czarny, Michał Bijak, Elżbieta Miller, Tomasz Śliwiński, Janusz Szemraj, Joanna Saluk-Bijak

**Affiliations:** ^1^Department of General Biochemistry, Faculty of Biology and Environmental Protection, University of Lodz, Pomorska 141/143, Lodz, Poland; ^2^Department of Medical Biochemistry, Medical University of Lodz, Mazowiecka 6/8, Lodz, Poland; ^3^Department of Physical Medicine, Medical University of Lodz, Pl. Hallera 1, Lodz, Poland; ^4^Neurorehabilitation Ward, III General Hospital in Lodz, Milionowa 14, Lodz, Poland; ^5^Department of Molecular Genetics, Laboratory of Medical Genetics, Faculty of Biology and Environmental Protection, University of Lodz, Lodz, Pomorska 141/143, Lodz, Poland

## Abstract

Nitric oxide (NO) is one of the most important signal molecules, involved in both physiological and pathological processes. As a neurotransmitter in the central nervous system, NO regulates cerebral blood flow, neurogenesis, and synaptic plasticity. The aim of our study was to investigate the effect of the extremely low-frequency electromagnetic field (ELF-EMF) on generation and metabolism of NO, as a neurotransmitter, in the rehabilitation of poststroke patients. Forty-eight patients were divided into two groups: ELF-EMF and non-ELF-EMF. Both groups underwent the same 4-week rehabilitation program. Additionally, the ELF-EMF group was exposed to an extremely low-frequency electromagnetic field of 40 Hz, 7 mT, for 15 min/day. Levels of 3-nitrotyrosine, nitrate/nitrite, and TNF*α* in plasma samples were measured, and NOS2 expression was determined in whole blood samples. Functional status was evaluated before and after a series of treatments, using the Activity Daily Living, Geriatric Depression Scale, and Mini-Mental State Examination. We observed that application of ELF-EMF significantly increased 3-nitrotyrosine and nitrate/nitrite levels, while expression of NOS2 was insignificantly decreased in both groups. The results also show that ELF-EMF treatments improved functional and mental status. We conclude that ELF-EMF therapy is capable of promoting recovery in poststroke patients.

## 1. Introduction

Cardiovascular diseases, including ischemic stroke (IS), are a serious problem of the modern age, killing 4 million people each year in Europe [1]. Stroke is caused by ischemia of brain tissue. Brain structure damage occurring during ischemia/reperfusion is due to the generation of significant amounts of reactive oxygen species and inflammatory mediators [2]. Damage to brain tissue as a result of a stroke cannot be undone. However, the most important part of poststroke therapy is immediate and long-term rehabilitation, considering the enormous plasticity of the brain [3]. Although extremely low-frequency electromagnetic field (ELF-EMF) therapy is not a standard treatment in the poststroke rehabilitation, some authors suggest its increased positive effect on patients [4]. ELF-EMF treatment is based on regeneration, osteogenesis, analgesics, and anti-inflammatory action. Its biological effect is related to processes of ion transport, cell proliferation, apoptosis, protein synthesis, and changes in the transmission of cellular signals [5]. The regenerative and cytoprotective effect of ELF-EMF is based on mechanism associated with nitric oxide induction, collateral blood flow, opioids, and heat shock proteins [6].

Nitric oxide (NO) is an unstable, colourless, water-soluble gas with a short half-life (3–6 sec). The compound has one unpaired electron, which makes it a highly reactive free radical. It is characterized by the multiplicity of action in the body, in both physiological and pathological conditions [7]. Synthesis of NO in the organism is catalysed by nitric oxide synthase (NOS), occurring in three isoforms: neuronal (nNOS), inducible (iNOS), and endothelial (eNOS), encoded by different genes whose expression is subject to varying regulation. The constituent isoforms of NOS are eNOS and nNOS, whose activity is associated with concentration of calcium ions and the level of calmodulin in a cell, as well as with hypoxia, physical activity, and the level of certain hormones, that is, oestrogens [8]. In contrast, because it is closely related with the calmodulin, iNOS does not require a high concentration of calcium ions but is regulated by various endogenous and exogenous proinflammatory factors [9].

The two-stage synthesis of NO consists of the oxidation of L-arginine to N*ω*-hydroxy-L-arginine and, under the influence of NOS and oxygen, formation of L-citrulline and release of NO. All isoforms of NOS require the same cofactors: nicotinamide adenine dinucleotide phosphate (NADPH), flavin mononucleotide (FMN), flavin adenine dinucleotide (FAD), tetrahydrobiopterin (BH4), iron protoporphyrin IX (heme), and O_2_ [7].

Nitric oxide is one of the most important signal molecules, involved in both physiological and pathological processes. One of the major functions of NO is as a potent vasodilation, increasing the blood flow and regulation of blood pressure, which has been used in clinical practice for many years. Deficiency of this compound is observed in various disorders of many systems: cardiovascular, gastrointestinal, respiratory, and genitourinary [10]. The beneficial effects of NO lie in its platelet inhibition, macrophage cytotoxicity (antibacterial, antiviral, and antiparasitic), and protection of the mucosal lining of the digestive system. On the other hand, excessive expression of iNOS can be disadvantageous, for example, during sepsis. The adverse action of NO is associated with the production of superoxide anions and subsequent generation of peroxynitrite and hydroxyl radicals, which are highly toxic [11].

In the central nervous system, NO as a neurotransmitter regulates cerebral blood flow, as well as neurogenesis and synaptic plasticity. Furthermore, neuronal death is caused by high concentrations of NO by caspase-dependent apoptosis process and promotion of inflammation. Elevated levels of nitric oxide promote necrosis by energy depletion. On the basis of these mechanisms, NO is involved in the etiology of many neurological diseases, such as major depression, schizophrenia, epilepsy, anxiety, and drug addiction [12].

Our study was designed to investigate the effect of ELF-EMF on the metabolism of NO, as a signal molecule in the central nervous system, in the rehabilitation of acute poststroke patients.

## 2. Materials and Methods

### 2.1. Blood Sample Collection

Blood samples were collected into CPDA_1_-containing tubes (Sarstedt, Nümbrecht, Germany). Immediately upon doing so, a portion of the sample was frozen at −80°C and the rest of the samples centrifuged to isolate the plasma (15 min, 1500*g*) at 25°C. Blood samples were collected twice, at an interval of 14 days before and after a standard 10 sessions of therapy. For additional analysis of 3-nitrotyrosine levels, the blood samples were collected three times, at an interval of 28 days: before treatment, after 10 treatments, and after 20 treatments. All blood samples were taken in the morning (between 7 am and 9 am) under patient fasting condition and stored using the same protocol.

### 2.2. Subject Presentation

**Figure 1:**
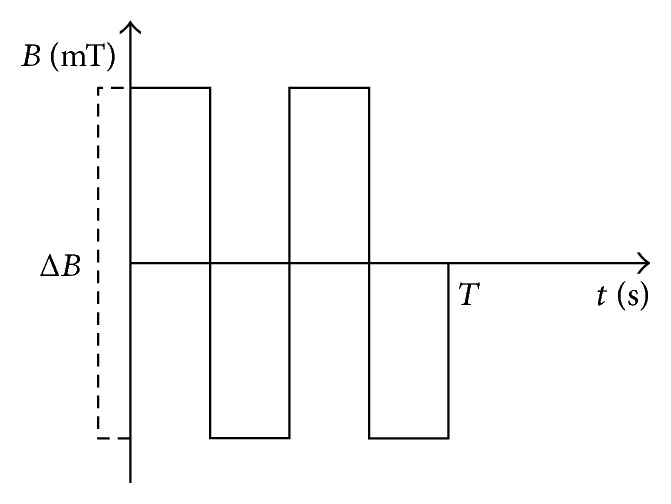
ELF-EMF description. Δ*B* = 5 mT; *T* = 1.3 sec.

Forty-eight poststroke patients were enrolled in the study. Participants were randomly divided into two groups: ELF-EMF (*n* = 25) and non-ELF-EMF (*n* = 23). Patients with metal and/or electronic implants (pacemakers, etc.) were excluded from the ELF-EMF group, for safety reasons. The ELF-EMF group had already undergone ELF-EMF therapy with specific parameters (40 Hz frequency, magnetic induction of 5 mT (Δ*B*), rectangular and bipolar waveforms) (Figure 1), which was conducted using a Magnetronic MF10 generator (EiE Elektronika i Elektromedycyna, Otwock, Poland). The parameters were selected on the basis of the fact that low-intensity stimuli improve the vital functions of the body. In addition, rectangular pulses are more intense than sinusoidal and trapezoid, while bipolar pulses show more range of changes than unipolar pulses [13]. The ELF-EMF and non-ELF-EMF groups were treated for the same amount of time (15 minutes). The non-ELF-EMF subjects were given only sham exposure. The pelvic girdle of the patients was exposed to the electromagnetic field, because exposure of the head to ELF-EMF can affect the activation of the epilepsy focus in the brain. The same therapeutic program was used for both subject groups. This consisted of aerobic exercise (30 min), neurophysiological routines (60 min), and psychological therapy (15 min). Poststroke patients with moderate stroke severity according to NIHSS scores of 4.9 ± 3.1 in the ELF-EMF group (aged 48.8 ± 7.7) and 5.4 ± 2.9 (aged 44.8 ± 8.0) in the non-ELF-EMF group were enrolled in the study.  Table 1 shows the clinical and demographic characteristics. Participants with haemorrhagic stroke, dementia, chronic or significant acute inflammatory factors, decreased consciousness, and/or neurological illness other than stroke in their medical prestroke history were excluded. The subjects had undergone neurorehabilitation for 4 weeks in Neurorehabilitation Ward III of the General Hospital in Lodz, Poland, as well as internal and neurological examinations. The Bioethics Committee of the Faculty of Biology and Environmental Protection of The University of Lodz, Poland, approved the protocol with resolution numbers 28/KBBN-UŁ/II/2015 and 13/KBBN-UŁ/II/2016. All participants provided written informed consent prior to participation. Depression was screened in both groups using the Geriatric Depression Scale (GDS). Cognitive status was estimated in a Mini-Mental State Examination (MMSE), and functional status using the Barthel Index of Activities of Daily Living (ADL). The GDS, ADL, and MMSE were administered either on the same day as the blood sampling or on the afternoon before.

### 2.3. Magnetronic MF10 Devices

ELF-EMF therapy was performed by a Magnetronic MF10 generator as per accepted guidelines. This device is able to produce pulses in rectangular, trapezoid, and sinusoidal shapes. The pulses were applied using an AS-550 applicator (EiE, Otwock, Poland), which has the following properties: 550 mm in diameter, 270 mm in length, and 5 layers of 187 turns of 1.45 mm twin-parallel wires. Magnetic induction was set at 5 mT. The electromagnetic field intensity was not uniformed; its distribution is vertical, while the induction coils are set horizontally. Induction of the electromagnetic field of 5 mT is present at the geometric center of the applicator, and the value increases in the proximity to the surface about 7 mT. Other factors that could affect EMF were eliminated (electronic measuring instruments occurring in rehabilitation room and other electronic equipment).

### 2.4. Immunodetection of 3-Nitrotyrosine by c-ELISA

Levels of 3-NT-containing proteins in plasma were determined using a modified c-ELISA method, as described by Khan et al. [14]. 96-well microtiter plates were coated with nitro-fibrinogen (nitro-Fg) (1 mg/mL) and kept overnight at 4°C. Concentrations of nitrated proteins inhibiting the binding of anti-nitrotyrosine antibodies were assessed from the standard curve (10–100 nM nitro-Fg equivalents) and expressed as nitro-Fg equivalents [15].

### 2.5. Nitrate/Nitrite Estimation

Plasma samples were diluted twice before the measurement of nitrate/nitrite concentration using a Nitrate/Nitrite Colorimetric Assay Kit (Cayman Chemical Company, USA), based on the two-step Griess method. In the first step, the nitrate is converted to nitrite with nitrate reductase, while in the second step, after addition of the Griess reagent, the nitrite is converted to a deep purple azo compound. The absorbance measurement was performed at 540 nm in a 96-well microplate reader (SPECTROstarNano, BMG Labtech, Ortenberg, Germany) [16].

### 2.6. Determination of *NOS2* Expression in Whole Blood Samples

RNA was isolated from the frozen whole blood samples (−80°C), in accordance with the manufacturer's protocol using TRI Reagent® (Sigma-Aldrich, USA). The aqueous phase was purified in accordance with the manufacturer's protocol using an InviTrap Spin Universal RNA Mini Kit (Stratec Biomedical Systems, Germany). The purity and quantity of isolated RNA were assessed using a Synergy HTX Multi-Mode Microplate Reader equipped with a Take3 Micro-Volume Plate and connected to a PC running Gen5 Software (BioTek Instruments Inc., Winooski, VT, USA). Isolated RNA (20 ng/*μ*L) was transcribed onto cDNA with a High-Capacity cDNA Reverse Transcription Kit (Applied Biosystems™, Waltham, MA, USA). Quantitative assays were executed using a TaqMan Hs01075529_m1 probe for human *NOS2* genes and an Hs02786624_g1 for endogenous control, which was *GAPDH* (Life Technologies). Reactions were carried out using a TaqMan Universal Master Mix II, without UNG (Life Technologies) in a BioRad CFX96 real-time PCR system (BioRad Laboratories, Hercules, CA, USA), all in accordance with the manufacturers' protocols. Relative expression of *NOS2* was obtained using the equation 2^−ΔCt^, where ΔCt is the threshold cycle (Ct) value for the target gene minus Ct values obtained for the housekeeping gene *GAPDH* [17].

### 2.7. Determination of TNF*α*

Measurements of human tumour necrosis factor alpha (TNF*α*) in plasma samples were made with a Human TNF*α* ELISA development kit (MABTECH, Cincinnati, OH, USA), in accordance with the manufacturer's protocol. The combination of two coating antibodies (TNF3 and TNF4) were used for the analysis. The absorbance was measured at 450 nm, and TNF*α* concentration was expressed as pg/mL [18].

### 2.8. Data Analysis

Biochemical and clinical data were expressed as mean ± SD. All measurements were executed in duplicate. Output value (100%) was determined for each measured parameter of each patient before treatment. Data from tests performed on the same study subjects after therapy constituted a percentage of the output value. Percentage values were presented as mean ± SD. Statistical analyses were performed using the Statistica 12 statistical software (StaftSoft Inc.). A Shapiro-Wilk test was used to analyse for normality. A paired Student *t*-test was used to the calculate differences between the values obtained for subjects before and after therapy, whereas unpaired Student *t*-test or Mann–Whitney *U* tests were used to determine differences between the ELF-EMF and non-ELF-EMF groups. *p* values of 0.05 were accepted as statistically significant for all analyses.

## 3. Results

**Figure 2:**
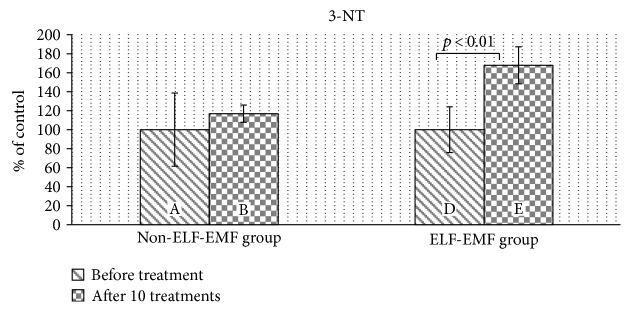
The comparison of 3-NT levels in plasma proteins obtained from the ELF-EMF group versus those from the non-ELF-EMF group. Statistical significance between the ELF-EMF and non-ELF-EMF groups: B versus D (*p* < 0.05).

**Figure 3:**
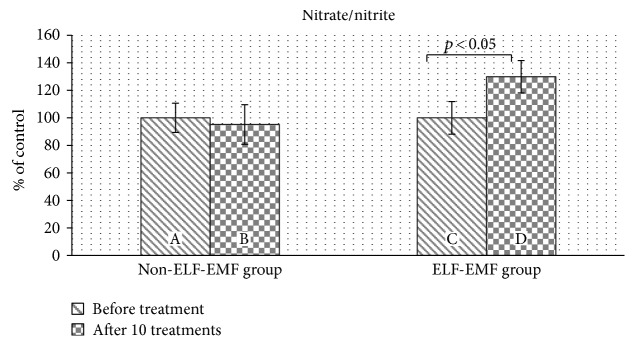
The comparison of nitrate/nitrite levels in plasma proteins obtained from the ELF-EMF group versus those from the non-ELF-EMF group. Statistical significance between ELF-EMF and non-ELF-EMF groups: B versus D (*p* < 0.05).

Our comparative analysis demonstrated an increased level of 3-nitrotyrosine (3-NT) (*p* < 0.05) (Figure 2) and an elevated nitrate/nitrite concentration (*p* < 0.01) (Figure 3) in the plasma of patients from the ELF-EMF group. The gain in the 3-NT level was significantly higher with an increased amount of sessions (Figure 2). In the non-ELF-EMF group, we saw that the effect of rehabilitation on nitrative stress was largely weaker and not statistically significant (*p* > 0.05) (Figures 2 and 3). The 3-NT level increased more in the ELF-EMF group than in the non-ELF-EMF after 10 treatments (68% versus 17%, *p* < 0.05) (Figure 2). The level of nitrate/nitrite in the non-ELF-EMF group even decreased after 10 treatments (although not statistically significantly) (Figure 3).

**Figure 4:**
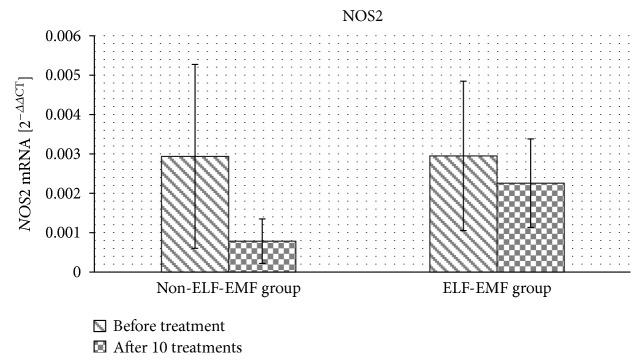
The comparison of *NOS2* mRNA expression obtained from the ELF-EMF group versus that from the non-ELF-EMF group.

In the next set of experiments, we determined the effect of magnetotherapy on gene expression in the whole blood samples of *NOS2* mRNA. Its expression was unmeasurable in 35% of subjects from both the ELF-EMF and non-ELF-EMF groups. We observed a statistically insignificant decrease in the level of *NOS2* mRNA expression after treatment in both the ELF-EMF and non-ELF-EMF groups (Figure 4).

**Figure 5:**
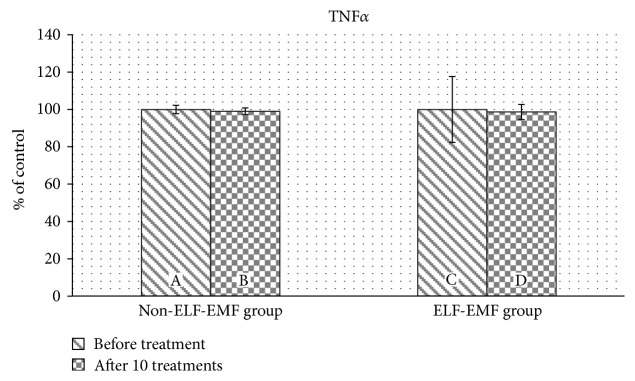
The comparison of TNF*α* levels in plasma proteins obtained from the ELF-EMF group versus those from the non-ELF-EMF group.

Subsequently, we determined the concentration of proinflammatory cytokine TNF*α*. We found that the concentration of TNF*α* was comparable before treatment in both the ELF-EMF and non-ELF-EMF-groups. The cytokine level did not change in either groups after rehabilitation (Figure 5).

The ADL, MMSE, and GDS were used to evaluate the functional and mental status of poststroke patients undergoing rehabilitation. We demonstrated that treatment using ELF-EMF improves their clinical parameters, particularly in cognitive and psychosomatic functions.

Motor abilities estimated by ADL score changed at similar levels in both groups, with the observed improvement being statistically significant in all rehabilitated patients (*p* < 0.001) (Table 2).

The baseline MMSE values before treatment in both groups were comparable, but statistically different (*p* < 0.05) after rehabilitation. After 2 weeks of rehabilitation, MMSE parameters improved markedly in the ELF-EMF group (*p* = 0.002), while a small increase in the non-ELF-EMF group was not statistically significant (*p* = 0.2) (Table 2).

Depression syndrome expressed by GDS improved significantly in both groups after rehabilitation. However, the ΔGDS value reached about a 60% lower result in the ELF-EMF group than in the non-ELF-EMF group (*p* = 0.018), starting from a similar base level in both groups (*p* > 0.05) (Table 2).

## 4. Discussion

In this study, we provide the evidence that application of extremely low-frequency electromagnetic field increases nitric oxide generation and its metabolism, as well as improving the effectiveness of poststroke ischemic patients' treatments.

Ischemic stroke is one of the major causes of morbidity and mortality in the world's population and is one of the main causes of long-term disability. The mechanisms of neurological function recovery after brain injury associated with neuroplasticity (cortical reorganization) are still insufficiently understood. Poststroke neurorehabilitation is designed to provide external stimuli, improving the effectiveness of compensatory plasticity [19].

In the central nervous system, NO is both a pre- and postsynaptic signal molecule. The activity of NO is associated with a cGMP-mediated signalling cascade. The presynaptic excitatory action of NO is related to the phosphorylation of synaptophysin by the cGMP-dependent protein kinase G (PKG) pathway and the subsequent potentates of glutamatergic neurotransmission [20]. On the other hand, NO causes a neurotransmission inhibition through gamma-aminobutyric acid- (GABA-) ergic synaptic communication. It is associated with ion exchange and regulation of membrane excitation [21, 22]. Moreover, NO as an important vasodilation factor mediates neurovascular coupling. The enlargement of vessel diameter is caused by increasing metabolic consumption as a result of neuronal activity. Neurovascular coupling maintains functional and structural brain integrity [23].

This study was designed to investigate the impact of ELF-EMF on the metabolism of nitric oxide in the rehabilitation of acute poststroke patients.

In our study, we demonstrate that poststroke rehabilitation increases the level of 3-NT and nitrate/nitrite concentrations. Due to its vasodilating and proangiogenic effects, NO serves as a protective function during cerebral ischemia. Su et al. investigated the role of simvastatin-regulated TRPV1 receptors (transient receptor potential vanilloid type 1) in NO bioavailability, activation of eNOS, and angiogenesis in mice. They demonstrated that simvastatin causes an influx of calcium ions through the TRPV1-TRPA1 (transient receptor potential ankyrin 1) pathway, which then causes activation of CaMKII (Ca^2+^/calmodulin-dependent protein kinase II). This then enhances the formation of the TRPV1-eNOS complex, which also includes CaMKII, AMPK (5′AMP-activated protein kinase), and Akt (protein kinase B), which leads to activation of eNOS, production of NO, and thus the promotion of endothelial angiogenesis [24]. There have been numerous reports of the protective effects of NO against inflammation and oxidative stress [25]. Transgenic eNOS-deficient mice demonstrated a more extensive infarct of the middle cerebral artery (MCA), compared to controls [26]. NO effects on the regulation of endothelial integrity, anti-inflammatory and anti-apoptotic effects, as well as maintenance of cerebral blood flow, inhibition of platelet aggregation, and reduction of leukocyte adhesion [25, 27]. Khan et al. studied structurally different NO donors as agents of cerebrovascular protection in experimentally induced stroke in rats. They showed that NO donors promote cerebral blood flow through S-nitrosylation and may be an effective drug for acute stroke [28, 29].

Furthermore, Greco et al. proved the protective effect of nitroglycerin (donors of NO) on cerebral damage induced by MCA occlusion in Wistar rats. They observed a significant reduction in stroke volume in preinjected rats compared to their control group, which confirms the protective effect of nitroglycerin *in vivo*. They speculated that the mechanism of action is associated with the generation of a complex chain of phenomena, triggering activation of apoptosis and subsequent activation of antiapoptotic responses [30].

The biological action of ELF-EMF is still being investigated. It is suggested that ELF-EMF has an impact on the physicochemical properties of water, the liquid crystal structure generated by cholesterol, and its derivatives [31, 32]. Changes in ion balance caused by ELF-EMF appeal to the structure of tissue with piezoelectric and magnetostrictive properties, free radicals, diamagnetic molecules, and uncompensated magnetic spins of paramagnetic elements [33]. Therefore, ELF-EMF causes depolarization of cells having the ability to spontaneously depolarize, predominantly through Ca^2+^ influx [34]. In our previous study, we investigated the effect of ELF-EMF on oxidative stress in patients after ischemic stroke. We demonstrated that ELF-EMF causes activation of antioxidant enzymes [35], which leads to reduction of the oxidative modification of plasma protein (this is detailed in an article published in *Advances in Clinical and Experimental Medicine*). As a highly reactive molecule, NO can also regulate the level of oxidative stress. Through the covalent interaction, NO influences the activity of various enzymes. Mechanisms of this modulation can be varied: NO reacts with coenzymes and active centers containing metal ions and interacts with cysteine residues of proteins [36].

In the current study, we observed that in the ELF-EMF group, the level of plasma 3-NT was increased (Figure 2). The formation of 3-NT in protein molecules occurs *in vivo* by the action of nitrating agents on the polypeptide chain. The formation of 3-NT is mainly attributed to NO and superoxide anions (O_2_^−^˙), which react rapidly to form peroxynitrite (ONOO^−^). This is one of the major oxidizing and nitrating agents produced *in vivo* in acute and chronic inflammation, as well as in ischemia/reperfusion. Endothelial cells, macrophages, and neutrophils release large amounts of NO and O_2_^−^. Thus, increased amounts of NO contribute to the creation of 3-NT [37].

To investigate the effect of ELF-EMF on NO metabolism, we determined nitrate/nitrite concentrations in plasma. We showed that in the ELF-EMF group, the level of nitrate/nitrite compounds in plasma increased after treatment (Figure 3), and these results correspond with the data presented by Chung et al. [38]. The authors investigated the effects of ELF-EMF (60 Hz, 2 mT) on the level of NO, biogenic amines, and amino acid neurotransmitters in the hippocampus, cortex, thalamus, cerebellum, and striatum in rats. They found a significant increase in NO concentration in the hippocampus, thalamus, and striatum. Moreover, ELF-EMF also caused a change in the level of biogenic amines and amino acid neurotransmitters in the brain. However, the observed effect and range were different, depending on the brain area. Balind et al. determined the effect of ELF-EMF (50 Hz, 0.5 mT) on oxidative stress in gerbils with induced cerebral ischemia. They measured the level of NO using the Griess reagent and showed an increased level of NO, provoked by electromagnetic fields. Moreover, ELF-EMF reduces oxidative stress generated during cerebral ischemia, thus leading to a decrease in the damaged brain tissue [39].

NO is produced from L-arginine with the involvement of nitric oxide synthase. Three NOS isoforms are expressed in different tissues. Although, in the blood, only NOS2 is expressed, in 35% of the subjects in both the ELF-EMF and non-ELF-EMF groups, mRNA expression of *NOS2* was under detection. In the remaining patients, the expression of *NOS2* had not significantly changed after treatment. The NOS2 gene in fact encodes for iNOS, which is primarily activated during inflammation. In order to exclude deeper inflammation, we measured the concentration of TNF*α*, one of the main proinflammatory cytokines. TNF*α* is a pleiotropic cytokine that is involved in nearly all phenomena of inflammatory responses: initiating chemokine synthesis, promoting the expression of adhesion molecules, promoting the maturation of dendritic cells, and inducing the production of inflammatory mediators and other proinflammatory cytokines [40]. TNF*α* stimulates collagenase synthesis in synovial fibroblasts and synovial cartilage chondrocytes and activates osteoclasts, leading to joint cartilage damage, hypertrophy, bone resorption and erosion, and angiogenesis. It also activates monocytes and macrophages, enhancing their cytotoxicity and stimulating cytokine production. Chemokines and growth factors are responsible for T cell proliferation, proliferation and differentiation of B lymphocytes, and the release of inflammatory cytokines by the lymphocytes. Moreover, in the hypothalamus, TNF*α* stimulates prostaglandin E and IL-1 synthesis [41]. Pena-Philippides et al. investigated the effect of pulsed electromagnetic fields on injury size and neuroinflammation in mice after middle cerebral artery occlusion (MCAO). They found, using magnetic resonance imaging (MRI), that EMF reduced infarct size, as well as changed expression of genes encoding pro- and anti-inflammatory cytokines in the hemisphere with ischemic injury. After EMF exposure, genes encoding IL-1*α* and TNF superfamily were downregulated, while *IL-10* expression was upregulated. Thus, the authors suggested that application of EMF to poststroke patients could have been beneficial through anti-inflammatory effect and reduction of injury size [42].

On the basis of our results, we suggest that the observed increase in NO level is associated with nNOS and/or eNOS activities, but not with iNOS expression. Our research is consistent with evidence shown by Cho et al., who established that ELF-EMF (60 Hz, 2 mT) increased the expression and activation of nNOS in rat brains [43].

The activities of nNOS and eNOS depend on calcium ions. There are many reports that the biological effect of ELF-EMF is related to the control of calcium channels [44–48]. In view of these findings, the observed mechanism of increased NO generation and metabolism may be associated with calcium-ion flux.

Additionally, we noticed that ELF-EMF treatment enhances the effectiveness of poststroke rehabilitation (Table 2). Some researchers suggest that electromagnetic fields have a beneficial effect on ischemic/reperfusion injury, and in some places, therapeutic programs using ELF-EMF are considered to be standard therapy for poststroke patients [49, 50]. The beneficial effects of ELF-EMF include the following: improvement in the transport of cellular and mitochondrial membranes; normalization of blood rheological values; counteraction of tissue oxidation; intensification of regenerative processes; stimulation of axon growth in undamaged neurons; intensification of neuronal dissociation and differentiation; reduction of stress-induced emotional reactions and free radicals; acceleration of the return of fibre function in functional disorders; reduction of periapical scarring; and increase of the level of energetic substances in the brain tissue and erythrocytes [48–53]. Grant et al. estimated the impact of low-frequency pulsed electromagnetic field on cerebral ischemia in rabbit. They observed using MRI that exposure to electromagnetic field caused extenuation of cortical ischemia oedema and reduction of neuronal injury in cortical area [54].

In conclusion, ELF-EMF therapy increases the metabolism and generation of NO, which has both neuroprotective and cytotoxic properties. An increase in NO level is probably associated with nNOS and/or eNOS activities, but not with iNOS expression, which increases mainly during inflammation. We suggested that in poststroke patients, NO demonstrated a protective effect due to significant improvement in patient functional status. Thus, our studies promote the validity of this method in poststroke rehabilitation therapy.

## Figures and Tables

**Table 1:** Clinical demographic characteristics.

		Control *n* = 23	Study group *n* = 25	*p*
Demographics	Age (mean ± SD)	44.8 ± 7.7	48.0 ± 8.0	0.84
	Sex: man versus female (%)	48 versus 52	60 versus 40	0.27
	Living alone (%)	32.1	34.2	0.59

Vascular risk	Hypertension (%)	97.3	98.5	0.07
	Diabetes (%)	31.4	39.2	0.21
	Dyslipidemia (%)	78.8	72.2	0.7
	BMI ≥ 30 (%)	21	34	0.78

Concomitant medications	Antidepressants (%)	29	34	0.5
	ASA (%)	70	65	0.42
	NSAID (%)	25	27	0.8

Stroke characteristics	Weeks since stroke (mean ± SD)	3.9 ± 0.6	3.2 ± 0.4	
	NIHSS scores (mean ± SD)	5.4 ± 2.9	4.9 ± 3.1	
	ADL (mean ± SD)	8.89 ± 2.87	9.95 ± 2.35	0.22

Lesion location	Anterior (*n*)	3	5	
	Posterior (*n*)	7	6	
	Intermediate (*n*)	13	14	

Lesion side	Left (*n*)	15	13	
	Right (*n*)	8	12	

**Table 2:** Clinical parameters: ADL, MMSE, and GDS measured in the ELF-EMF and non-ELF-EMF groups. Data presented as the delta of a clinimetric scale before and after the standard series of treatments (ΔADL = the increase of ADL; ΔMMSE = the increase of MMSE; and ΔGDS = the decrease of GDS).

		Non-ELF-EMF group	ELF-EMF group	*p*
ADL	Before treatment	8.99	9.95	0.378
	After treatment	14.3	16.12	0.429
	Δ	5.31	6.17	0.194
	*p*	<0.001	<0.001	

MMSE	Before treatment	22.75	23.00	0.873
	After treatment	23.94	26.28	0.047
	Δ	1.19	3.28	0.036
	*p*	0.204	0.002	

GDS	Before treatment	11.38	13.00	0.054
	After treatment	9.13	6.72	0.049
	Δ	2.25	6.28	0.018
	*p*	0.009	0.001	
